# Comprehensive analysis of the renal transcriptional response to acute uranyl nitrate exposure

**DOI:** 10.1186/1471-2164-7-2

**Published:** 2006-01-11

**Authors:** Magali Taulan, Francois Paquet, Angel Argiles, Jacques Demaille, Marie-Catherine Romey

**Affiliations:** 1 Laboratoire de radiotoxicologie expérimentale, Institut de Radioprotection et de Sûreté Nucléaire, Site du Tricastin, BP 166 26702 Pierrelatte Cedex, France; 2 Laboratoire de Génétique Moléculaire, UPR 1142, Institut de Génétique Humaine, 141 Route de la Cardonille, 34396 Montpellier Cedex 05, France; 3 Laboratoire de Génomique Fonctionnelle, UPR 2580, Institut de Génétique Humaine, 141 rue de la Cardonille, 34396 Montpellier Cedex 5, France

## Abstract

**Background:**

Chemical and radiological toxicities related to uranium acute exposure have been widely studied in nuclear fuel workers and military personnel. It is well known that uranyl nitrate induces acute renal failure (ARF). However, the mechanisms of this metal-induced injury are not well defined at the molecular level.

**Results:**

Renal function and histology were assessed in mice receiving uranyl nitrate (UN(+)) and controls (UN(-)). To identify the genomic response to uranium exposure, serial analysis gene expression (SAGE) of the kidney was performed in both groups. Over 43,000 mRNA SAGE tags were sequenced. A selection of the differentially expressed transcripts was confirmed by real-time quantitative PCR and Western blotting. UN(+) animals developed renal failure and displayed the characteristic histological lesions of UN nephropathy. Of the >14,500 unique tags identified in both libraries, 224 had a modified expression level; they are known to participate in inflammation, ion transport, signal transduction, oxidative stress, apoptosis, metabolism, and catabolism. Several genes that were identified had not previously been evaluated within the context of toxic ARF such as translationally controlled tumor protein, insulin like growth factor binding protein 7 and ribosomal protein S29, all apoptosis related genes.

**Conclusion:**

We report a comprehensive description of the UN induced modifications in gene expression levels, including the identification of genes previously unrelated to ARF. The study of these genes and the metabolisms they control should improve our understanding of toxic ARF and enlighten on the molecular targets for potential therapeutic interventions.

## Background

Uranium (U) is a heavy metal, which has a wide range of uses that invariably carry an exposure risk for industrial workers as well as for the general population. After an accidental absorption of U compounds, the soluble form of U is carried in the blood, filtered by the glomerulus and partly excreted in the urine. Uranyl nitrate (UN)-induced nephropathy has been extensively studied in animal models. [1-3] It mainly involves the S3 segment of the proximal tubule [2].

Many studies on toxicant or ischemia-induced acute renal failure (ARF) have provided data demonstrating the participation of a wide range of compounds that are known to modulate several processes such as nephrogenesis, regenerative response and inflammation [4-11]. However, the bulk of knowledge on ARF at the molecular level, has been achieved with work that focused on a single molecule or pathway characterization [6,11].

The tremendous progress recently accomplished in biotechnology has made possible to analyze thousands of transcripts in a single experiment, thereby offering a powerful strategy in the study of transcriptome. Some studies have been already performed to analyze the genomic responses in various types of ARF (table 1) [12-17]. However, many aspects remain to be unraveled in ARF. Here, we described for the first time the renal toxicogenomics effects of UN-induced ARF *via *SAGE (Serial Analysis of Gene Expression) technology. The potential of this approach for the discovery of novel toxicant-induced gene expression alterations has been already highlight following UN-long term exposure [18]. In addition, the choice of SAGE versus microarray hybridization technique depends on several factors, such as the scope of the genetic screen, the number of samples, the amount of starting material and the availability of resources such as an automated DNA sequencer. Unlike microarray approaches, SAGE is an unbiased method in that it does not require a priori knowledge of genes of interest and is therefore not constrained by sequences on a chip. Furthermore, SAGE data contains expression information for every tag (gene) relative to every other tag in a given library and so requires minimal normalization and, since it generates immortal data, can be readily shared between laboratories. In addition, although both functional and histologic damages resulting from uranium exposure have been well established, little is known about molecular effects of UN contamination. Therefore, we chose SAGE approach to provide a comprehensive view of the molecular events involved in the early phase of UN-induced ARF. Our results demonstrate a variation in the expression rate of i) genes previously related to both, ischemic and toxic ARF (including UN-induced ARF), ii) genes that were known to be related to the various types of ARF, but not to participate in UN-induced ARF and iii) genes that were not known to participate in either type of ARF such as translationally controlled tumor protein (*TCTP*), insulin like growth factor binding protein 7 (*IGFBP7*) and ribosomal protein S29 (*Rps29*).

## Results

### Characterization of UN-induced ARF

All the animals included in the study survived at either UN or vehicle injections. As expected, only the animals receiving UN injections developed ARF. Tissue levels of uranium were significantly increased in UN(+) group compared to UN(-) group (15.8 ± 5.6 μg/g vs. 0.3 ± 0.9 μg/g, p < 0.001).

#### Renal function

**Figure 1:**
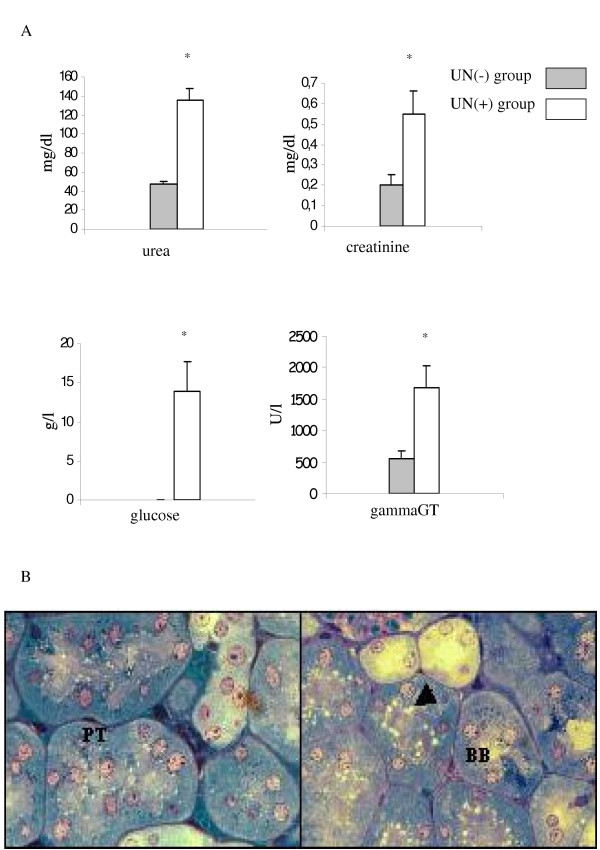
**Characterization of UN-induced ARF**. **(A) **Influence of UN on renal function. Blood levels of urea and creatinine and urinary levels of glucose and γGT are plotted. The bars represent the mean ± SEM. *p < 0.05 vs control. **(B) **Light micrographs of renal tissue from UN(-) (left panel) and UN(+) (right panel) group given 5 mg/kg UN intraperitoneally. Proximal tubules (PT), sectioned transversely, are lined by high cuboidal or pyramidal epithelium. A slight loss in the brush border (BB), and large vacuoles (arrow) in proximal tubular cells in comparison with control group are depicted (×40).

Urea and creatinine serum levels significantly increased in UN(+) group as early as day 2 (figure 1A). Urinary γGT also significantly increased and glucose was detected in UN(+) group (figure 1A).

#### Morphological changes

UN injected animals displayed an important tubular vacuolization and a focal loss of brush border in proximal tubular cells (figure 1B). The score of proximal tubules (PT) damage in outer stripe of outer medulla (S3 segments) compared to UN(-) tissue was increased as early as day 2 (between 25 and 50% of PT damaged). The overall structure of the kidney was preserved and glomerulae were intact in appearance. These findings are well in keeping with previous reports [19]. Because it is well known that changes in gene expression occur soon after a renal insult, we decided to examine the early response of an UN contamination. Although, the histological observations are not very convincing, we chose genome-wide gene-expression approach because it allows more sensitive end points to evaluate renal impact of UN treatment.

### Global gene expression in UN-induced ARF

The sequence analysis of ≈ 43,000 transcripts from the kidneys of UN(-) and UN(+) tags was performed. The vast majority of these tags represent distinct transcripts. However, some tags, especially those detected only once, may result from PCR or sequencing errors [20]. More than 7,900 and 6,800 distinct tags were obtained in UN(-) and UN(+) animals, respectively.

The good quality of the obtained libraries could be confirmed based on the previously reported renal transcriptomes (table 1) [21-27]. As expected, we predominantly found, tags specific for proximal tubule, as it represents the bulk of kidney's weight. Further, a large fraction of the most abundant tags matches with widely expressed mitochondrial genes or ribosomal proteins. The high levels of mitochondrial DNA-encoded transcripts are consistent with the high-energy demand of the kidney and have been previously noted in kidney SAGE libraries by other investigators [26,27]. In addition, in agreement with previous data, the most frequently observed tags corresponded to glutathione peroxidase3 (*GPx3*) (2.1%) and *KAP *(1.4%) genes [26].

**Figure 2:**
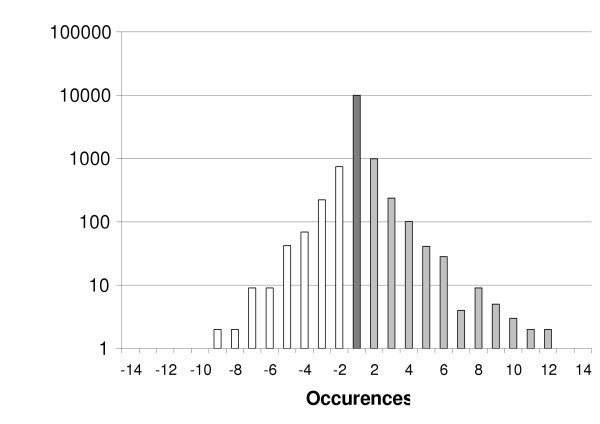
**Summary of the SAGE data**. The ratio of the number of occurrences for each given unique tag in both libraries (UN (-) and UN (+)) has been calculated. Tags have been grouped according to their expression ratio (X-Axis). The frequency (Y-Axis) plot of this ratio shows a Gaussian distribution.

Data analyses showed that 2,518 tags (61.4%) match with known genes, 223 retained tags (5.4%) do not match with characterized mouse genes or cDNAs. When considering EST databases, 347 tags (8.5%) match with anonymous sequences. One thousand and sixteen tags (24.7%) are assigned to RIKEN sequence in the UniGene database. Monte Carlo simulations were used to determine the statistical significance of differentially expressed genes from both libraries (figure 2). As expected, the expression level was unmodified after UN injection for the majority of transcripts. Interestingly, 224 transcripts were significantly over-expressed (116) or under-expressed (108) in UN(+) animals (listed in table 3). After exclusion of tags matching mitochondrial sequences, and those with multiple and non-reliable matches, only tags with a significant expression change (p-values<0.05) were retained and arbitrarily grouped according to their function. SAGE analysis revealed an overexpression of genes related to the release of inflammatory mediators, such as the secreted phosphoprotein 1 (S*pp1 *or O*pn *7/104), *Umod *(which encodes for the Tamm-Horsfall protein, 23/69), lectin galactose binding (*gal-3*, 1/13), tumor necrosis factor alpha-induced protein (12/21) and *Tctp *(listed as translationally regulated transcript, 149/263) gene. Many genes involved in signaling were down-regulated such as hormonal receptors (growth hormone receptor, 15/3; parathyroid hormone receptor, 17/8) and transcripts encoding for chaperone proteins including heat shock 70 kD protein 8 (*Hsp70*, 52/29) and heat shock protein, 60 kDa (13/4). *Igfbp7 *(45/85), also known as *Igfbp-rP1/mac25 *was found to be increased. The UN-induced transcripts mainly consist of gene encoding proteins associated with lipid metabolism (hydroxysteroid dehydrogenase, 25/10 and alcohol dehydrogenase, 7/1), amino acid metabolism (glutamate dehydrogenase, 4/16 and *Odc*, 53/14) and carbohydrate metabolism (fructose bisphosphate, 25/8; glucose phosphate isomerase, 0/6). UN exposure also increased the expression of a number of genes related to oxidative process and detoxification, including cytochrome P450 (*Cyp4b1*, 16/7) that catalyses the oxidation of a wide variety of substrates [29]. Other enzymes, such as peroxiredoxin 1 (29/15), S*od1 *(19/6), peroxiredoxin 2 (1/8), thioredoxin-like 2 (2/9), ferritin light chain (32/62) were significantly under- or overexpressed. We also observed down-regulation of genes related with ion transporters including solute carrier family 22 (organic cation transporter)-like2 (57/23) and 34 (sodium phosphate), member1 (23/8) according with previous studies [29]. Numerous transcripts encoding for ribosomal proteins were up-regulated, such as *Fau *gene that encodes an ubiquitin-like protein fused to the ribosomal S30 protein (the cellular homolog of the fox sequence of the Finkel-Biskis-Reilly murine sarcoma virus (FBR-MuSV) [30], L9 (21/52), L19 (77/131), Large P1 (55/124) and S29 (37/81).

### Confirmation of SAGE data

#### Real-time quantitative PCR analysis

**Figure 3:**
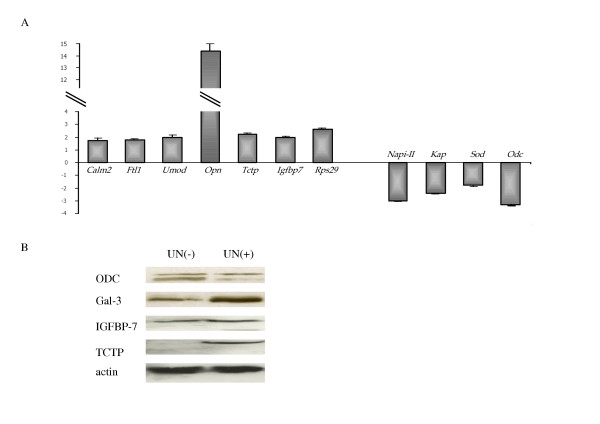
**Confirmation of SAGE data**. **(A). **Real-time RT-PCR analysis. The relative fold change of UN(+)/UN(-) is plotted. PCR analyses were performed on cDNA from UN(-) or UN(+) tissues. All values represent the mean ± SEM. *p < 0.05 vs control. **(B). **Western blot analysis of ODC, TCTP, IGFBP7 and Gal-3 proteins in UN(-) and UN(+) tissues. Fifty micrograms of total cell protein were loaded per lane on a 10% SDS-polyacrylamide gel, and ODC, TCTP, IGFBP7 and Gal-3 were detected by immunoblotting with antibodies against the respective proteins. As a control for proteins loading in SDS-PAGE, an anti-β-actin antibody was used.

We performed real-time quantitative PCR analyses of eleven selected genes to confirm the differential expression observed by SAGE. *Kap *was chosen because of its abundance in normal (1.4%) and in contaminated (0.38%) kidney. *Opn *was chosen because we observed an increase in its expression rate in UN-ARF and it had been previously related to uranyl acetate-induced ARF [31]. *Umod *was chosen as it was increased in our SAGE data while it was previously reported to decrease in ARF [16]. *Sod1*, *Odc*, Calmodulin 2 (*Calm*2), Solute carrier family 34 (sodium phosphate) member1 (*NaPi-II*) and Ferritin light chain 1 (*Ftl1*) were chosen as they were modified in ARF [3,17,29,32]. Results for all these transcripts corroborated the expression differences observed in our SAGE analysis (Figure 3A). *Tctp*, *Igfbp7 *and *Rps29 *were included in the RT PCR analysis since they were observed to vary in ARF for the first time in our study. Real-time confirmed the SAGE results, as it showed an increase in PCR expression of *Opn, Umod*, *Ftl1*, *Calm2*, *Tctp*, *Igfbp7 *and *Rps29 *and a decrease in expression of *kap*, *NaPi-II*, *Odc *and *Sod *in ARF induced by UN.

#### Western blotting

To determine whether the gene expression changes were apparent at the protein level, we performed Western blotting experiments. The choice of the selected protein depends on the interest of the protein and the ability to obtain certain antibodies. The protein products of three genes with increased expression level (Gal-3, IGFBP7 and TCTP) and one with decreased expression level (ODC) were selected. Expression level was evaluated using β-actin gene as reference gene. Immunoblot analysis confirmed the accuracy of the differences in expression level observed in our SAGE analysis (Figure 3B).

## Discussion

The kidney is a complex organ consisting of well-defined components that function in a highly coordinated fashion. When a segment of the functional unit of the kidney is altered, ARF may occur. Tubules are the most frequently damaged area in ARF. Some mechanisms responsible for tubular damage are already well described such as inflammation, apoptosis and oxidative stress. However, the molecular events involved in the cellular response to renal injury are not completely elucidated, neither those participating in inflammation, apoptosis and oxidative stress, nor those participating in other cell metabolisms that might also be involved in ARF. To obtain a holistic view of the molecular processes involved in nephrotoxic ARF, we studied the changes in the transcriptome of kidney after UN treatment.

Genome level transcriptional profiling of a given cell, tissue, or organ provides a map of interconnected molecular pathways and reflects coordinated response of their various components to a given physiological condition. Because Gene chips only measure the expression of genes represented on the chip in contrast to SAGE, in which the expression profile of the complete transcriptome can be mapped, we have used SAGE approach to identify renal molecular effects of acute exposure to uranium in mice. SAGE is a high-throughput method for quantitative evaluation of global gene expression [33]. Similarly to microarray technology, SAGE can be used to evaluate genome-wide transcriptional response of a given-cell, tissue, or organ to environmental stimuli. Nonetheless, whereas large numbers of samples may be analyzed more efficiently by using the microarray technique, SAGE does not depend on a priori gene knowledge and thus represents a truly unbiased technique.

More than 200 transcripts out of the >14,500 unique transcripts observed were differentially expressed in UN(+) and in UN(-) animals. The differentially regulated genes could be distributed into three groups: group 1 including those genes that had been previously identified to participate in all forms of ARF, group 2 which encloses those genes participating in ARF but not previously related to the UN-induced form of ARF, and group 3 that includes those genes that had not been previously suspected to participate in any form of ARF. All three groups of genes are relevant in our study, since the group 1 genes validate our system in relation to previous reports (they represent an appropriate internal positive control). Group 2 genes demonstrate that some of the molecules participating in UN-induced ARF are shared with other forms of ARF. Finally, group 3 genes provide evidence for involvement in ARF of newly identified genes.

Although, the genes identified by SAGE are informative on the possible endeavors that may be taken to extend our research on the understanding of UN-related ARF, supplementary studies with other techniques should follow. However, to choose the ways to focus our future efforts, it is capital to evaluate each gene that has been identified according to the metabolic systems it participates and the relevance it may have on the events observed in ARF. Following, we include a brief analysis of those genes retaining our attention on these grounds.

Among the group 1 genes, *Opn *is the most differentially expressed gene (15-fold increase). Osteopontin is involved in inflammation [6] and in proximal tubule regeneration [31], and has been proposed as a marker for renal injury [34]. Also among the genes of group 1, Cu, Zn-SOD, a key enzyme against oxidative stress, was underexpressed in UN-induced ARF, in agreement with previous data [3]. This description underlies the poorly studied molecular mechanism related to UN-induced ARF.

*Gal-3*, a gene of group 2, was up-regulated (1/13). Gal-3 is a pro-inflammatory molecule [35] containing in its sequence the asparagine-tryptophan-glycine-arginine motif, highly conserved in the BH1 domain of the Bcl-2, a well characterized suppressor of apoptosis [36]. A decrease in apoptosis is known to be related to an increase in mitochondrial integrity, a diminution in cytochrome C release, and caspase activation; three features that have been described for Gal-3 [36]. However, the mechanisms by which *Gal-3 *participates in UN-induced ARF are not known. Exploring this point seems of interest in ARF. Among the genes that had not been previously related to UN-ARF, we also noted metallothionein 2(MT-2) and Calm 2, known to be up-regulated after a heavy metal contamination [37,38]. The latter, a putative calcium transporter, is essential for mitotic progression and its up-regulation may be a response to possible direct metal effect [37]. The responses of MT-2 are not surprising, as it is known to participate in stress response. Despite many decades of research, the present physiologic function of MT has not been elucidated. However, some suggested functions attributed to this protein include detoxification of nonessential heavy metals (cadmium and mercury), homeostasis of zinc and copper, metal transfer, free-radical scavenger, and metal storage [38]. Another interesting gene of this group 2 is *Umod*. It encodes for Tamm-Horsfall protein and has been reported to be down-regulated in ischemia-induced ARF [16]. Our SAGE analysis surprisingly showed an up-regulation of *Umod *expression. This increase was also confirmed by our RT-PCR studies, suggesting that THP behaves differently in ischemia- and UN-induced ARF. THP has been related to inflammation and showed to participate in hyperuricemic nephropathy as well as in medullary cystic disease [39]. In addition, an up-regulation of UMOD has been observed in the progression of nephrolithiasis [40]. The similarity between renal stone formation and UN crystal deposition, as well as the putative role of THP in these forms of ARF certainly deserves further study. Another interesting family of genes is constituted by the NaPi-II transporters, which are mainly expressed on the brush border of PT and modulate tubular reabsorption of inorganic phosphate [29]. Their renal expression was decreased in UN(+) animals, in keeping with the tubular damage and increased γGT urinary levels. Also among the genes of group 2, ODC, the rate-limiting enzyme of polyamine biosynthesis, was down-regulated in UN-induced ARF. Studying a model of chronic renal failure, Fleck *et al. *also observed a decrease in *Odc *expression level 10 weeks after a single injection of UN [41]. We confirmed this observation following UN long-term exposure in mice [18]. Kramer *et al. *have obtained a depletion of polyamine pool using a specific biosynthetic enzyme inhibitor of ODC, resulting in p21-mediated G1 cell cycle arrest [42]. Therefore, the decrease in *Odc *mRNA level might be related to an arrest of cell cycle following UN treatment. The putative role of ODC and the participation of the cell cycle on UN induced ARF also appear important. Finally in this group 2, we observed a decrease in the expression level of *Kap *gene. While its expression in S1 and S2 segments is androgen dependent, no androgen is required for KAP expression in S3 segment. KAP is then observed in S3 of female and castrated males [43] and has been used as a S3 marker [26]. It participates in thyroid and growth hormone/insulin-like growth factor-1 axis, known to be involved in other forms of ARF.

The most novel part of our results concern group 3 genes: those that had not been previously related to ARF. Three genes were identified *Rps29*, *Igfbp7 *and *Tctp *and all of them are markedly up-regulated in UN induced ARF. RPS29 is a potent apoptosis-inducing agent [44], and its over expression further supports the participation of apoptosis in UN-induced ARF. IGFBP7 is a potential transcription factor [45] with a variable distribution along the renal tubular epithelium. It could be involved in the regulation of cell growth and differentiation [46]. In addition, it might have a tumor suppressor activity through the induction of cell senescence [46]. Therefore, the study of the participation of this protein in UN-induced ARF is also of interest. Finally, TCTP is a protein usually found in the cytoplasm of both normal and tumor cells lines. This finding provide additional evidence for an up-regulation of TCTP in animals treated by heavy metal exposure [47]. Interestingly, we also observed an over-expression after UN long-term ingestion [18]. However, it is the first time that enhanced *tctp *expression results from acute UN exposure. TCTP has been previously characterized as an inflammatory molecule with an IgE-dependent histamine-releasing capacity [48]. It was also described as an antiapoptotic protein [46]. TCTP, a tubulin-binding like protein is associated with microtubules in a cell cycle dependent manner [48] and is associated with components of the translational machinery [49]. Moreover, its expression is regulated by Ca^2+ ^[48]. Thus, TCTP is implicated in cell growth and differentiation, acute allergic response, apoptosis and other various processes [48,49] and may be a central protein in the phenomena observed in UN-induced ARF. More work will be necessary to establish whether TCTP might be suitable as diagnostic tool or for other medical application in connection with renal failure.

## Conclusion

Whereas the full potential of SAGE for gene expression profiling could not be exploited due to the difficulties in tag to gene assignment, one of the major strengths of SAGE is the electronic nature of the database, allowing further investigations and direct comparisons of libraries in silico by different investigators. Nevertheless, this first SAGE analysis lays the basis for furthers studies. Indeed, accumulation of additional data will increasingly facilitate the interpretation of results because bona fide tags will be distinguished from artifacts by being replicated and even polymorphic tags will eventually be defined and assigned to their corresponding transcripts. Despite these promising results, further investigations must be performed to increase the usefulness of transcript profiling in toxicology. A next step will be to address issues related to dose-response relationships and human exposure assessment. This will allow us to confirm whether observed gene expression changes are effectively indicative of toxic liabilities when standard parameters do not yet detect toxicity as previously reported in UN-long-term exposure [18].

**Figure 4:**
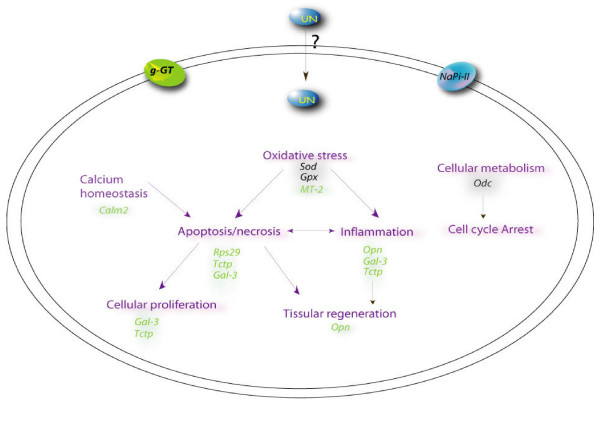
**Putative cellular pathways of UN-mediated nephrotoxicity**. The diagram depicts the putative pathways involved in UN-mediated toxicity. This schematic was assembled using the gene expression data from 2 days UN treatment and previous litterature. Up- or down-regulation is denoted by different colors (green and black, respectively). Mechanisms (in purple) such as oxidative stress, apoptosis, inflammation and perturbation of calcium homeostasis might contribute to uranium toxicity. Secondary compensatory mechanisms were evident as increases in tissue regeneration and cellular proliferation.

In this report, we elegantly demonstrated that UN-induced renal injury is associated with dramatic alterations in gene expression profile, as presented on the depicted drawing (figure 4). UN up- or down-regulates genes involved in inflammatory, in oxidative response, in protein and lipid metabolism, in androgenic response and in solute transporters. Changes in expression of a selected group of genes, known to participate in ischemic and toxic ARF (OPN and SOD) and also in genes known to be linked to ischemic or toxic ARF but not previously described in UN-induced ARF (KAP, UMOD, Calm2, Ftl1, NaPi-II and ODC), were confirmed at both mRNA and protein level (Gal-3 and ODC). Our SAGE analysis, in addition to showing the global changes in transcriptome, enabled us to identify new genes associated to ARF (e.g. TCTP, IGBFP7 and Rps29). This finding of potential pro- or anti-apoptotic up-regulated transcripts by SAGE analysis was confirmed by quantitative RT-PCR and immunoblot. Further global analyses of gene expression changes associated with different types of ARF will be necessary to define genes which are potential hallmarks of specific mechanisms of UN-renal injury and which are more general markers of renal damage. Apoptosis, cell cycle and inflammation appear as the main features in UN-induced ARF. Indeed, apoptosis has recently emerged as the major mechanism leading to early tubule cell death following ischemia-reperfusion injury and down-regulation of apoptosis may offer a novel therapeutic approach for the amelioration of ischemic or toxic renal injury. Further study of the genes implicated in these processes should shed light on our understanding of the physiopathology of toxic ARF; a mandatory step in identifying new markers and/or treatment targets of the disease.

## Methods

### Animals

Twenty male C57 Bl/6J mice, weighing 25–30 g (Harlan, France) were housed in light controlled rooms with 12-h periods of light and darkness and free access to food and water. They were randomly divided into two groups: controls (UN(-)) and Uranyl nitrate-treated (UN(+)) mice.

### Uranyl nitrate-induced acute renal failure

A single dose of 5 mg/Kg of Uranyl nitrate (Merck, France) in 0.9% NaCl, or the vehicle alone, was given intraperitoneally [1]. The animals were euthanized by ex-sanguination using cardiac puncture 48 hours after the injection to evaluate early renal injury [34]. As we did not perform a time-course, the time-point and concentration choices are based on prior knowledge [1,34]. Uranium residues were examined in samples of kidney using a kinetic phosphorescence analyzer (KPA) [50]. Serum creatinine and urea levels and urinary concentrations of glucose, gamma-glutamyltranspeptidase (γGT) were measured by routine methods.

### Sample preparation

The kidneys were either embedded in epon for morphological examination or snap-frozen in liquid nitrogen and then stored at -70°C until further study.

### Preparation of kidney samples for morphological examination

#### Preparation

Kidney specimens were cut into 1 mm cubes and fixed with 3% gluteraldehyde in aqueous solution. Following post-fixation, the cubes were dehydrated in ascending grades of ethanol and absolute ethanol for 1 h30, infiltrated with propylene oxide and propylene oxide-epoxy resin, and finally embedded in epon.

#### Morphology

For light microscopy, 1 μm sections were cut from tissue blocks using a Leica ultramicrotome. The sections were mounted on glass slides and stained with toluidine blue (1%), basic fuchsine dye solution (1%) and examined under a Zeiss optical microscope.

#### Score

Tubular injury was ranked according to tubular cells desquamation in grade 0 (normal tissue), grade 1, 2, 3, 4 (cells desquamation <25, 25–50, 50–75, 75–100% respectively).

### RNA isolation

Total RNAs, extracted from the renal tissue using the RNA isolation mini kit (Qiagen, France) were pooled and stored at -70°C until further study. The amount of total RNA was determined using a fluorescent nucleic acid stain (RiboGreen RNA Quantitation kit, Molecular Probes). The quality of the RNA was evaluated by measuring the 260:280-nm ratios and confirmed by visualization of intact 18S and 28S RNA bands after agarose gel electrophoresis. For SAGE analysis, in both treated and control groups, an equal amount of kidneys extracted RNAs from each mouse was pooled into a single sample. In some selected instances, RNA from a single animal was analyzed by real-time RT-PCR.

### Analysis of gene expression

Kidney libraries were generated from 50 μg of total RNA using I-SAGE kit (Invitrogen, France) following the manufacturer's instructions [51], adapted from initial description [33], and previously described [18]. SAGE data for the libraries described here are available at Gene Expression Omnibus [52]; accession nos. GSM 24251 and GSM 24255). In Results section, tags occurrence were arbitrarily noted as follow: tags occurrence in UN(-)/tags occurrence in UN(+).

### Real-time RT-PCR

Total RNAs (1 μg) from UN(-) and UN(+) renal tissue (extracted as described above *RNA isolation*) were used to generate cDNA using M-MLV-RT (Invitrogen) according the manufacturer's conditions. Primers and probes specifically designed for selected cDNA using PRIMER EXPRESS software version 2.0 (PE, Applied Biosystems), are listed in table 2. The ABI PRISM 7000 sequence detection system was used for detected real-time RT-PCR products with the SYBR Green I assay according to recommendations of the manufacturer (PE, Applied Biosystems). For three cases, in which we encountered difficulties with the SYBR Green I assay, we used the TaqMan probe assays (table 2). Each PCR reaction was optimized to ensure that a single band of the appropriate size was amplified and that no bands corresponding to genomic DNA amplification or primer-dimer pairs were present. The PCR cycling conditions were performed for all samples as follows: 50°C, 2 min for AmpErase UNG incubation, 95°C, 10 min for AmpliTaq Gold activation, and 40 cycles for the melting (95°C, 15 s) and annealing/extension (60°C for 1 min) steps. PCR reactions for each template were done in triplicate in 96-well plates. The comparative ΔΔCt method (PE, Applied Biosystems) was used to determine relative quantitation of gene expression for each gene compared to the HPRT control (listed in table 2).

### Western blotting

Fifty micrograms of whole proteins extracted from either UN(-) or UN(+) tissues were subjected to SDS-PAGE, and then electrotransferred onto nitrocellulose membranes. Quantitative protein and even transfer in each lane was verified by reversible protein staining of the membranes with 0.1% Ponceau S in 5% acetic acid. After blocking in 5% non-fat dry milk, 0.1% Tween 20 blots were incubated with specific antibodies against Gal-3 (1:100 dilution, Interchim), TCTP (1:5000 dilution, Santa Cruz), IGFBP-7 (1:5000 dilution, Santa Cruz), ODC (1:200 dilution, Sigma) and β-actin (1:2500 dilution, Sigma) ON at 4°C. The membranes were then washed and incubated with appropriate horseradish peroxidase conjugated secondary antibody at 1:15 000 (Sigma) for 1 h. All antibodies were diluted in PBS containing 5% non-fat dry milk. The membranes were reacted with chemiluminescence reagent ECL (Roche Diagnostic) as described by the manufacturer and subsequently exposed to Biomax photographic film (Kodak Corporation). The protein level of the actin housekeeping gene were assayed for internal control of protein loading.

## List of abbreviations

ARF: Acute Renal Failure

Calm2: Calmodulin 2

Ftl1: Ferritin light chain 1

Gal-3: Galectin-3

γGT: gamma-Glutamyltranspeptidase

GPx3: Glutathion Peroxidase 3

IGFBP7: Insulin like Growth Factor Binding Protein 7

KAP: Kidney Androgen regulated Protein

KPA: Kinetic Phosphorescence Analyzer

MT-2: Metallothionein 2

NaPi-II: Solute carrier family 34 (sodium phosphate member 1)

ODC: Ornithine Decarboxylase

OPN: Osteopontin

Rps29: Ribosomal protein S29

SAGE: Serial Analysis of Gene Expression

SOD: Superoxide Dismutase

TCTP: Translationally Controlled Tumor Protein

THP: Tamm-Horsfall Protein

U: Uranium

UMOD: Uromodulin

UN: Uranyl Nitrate

## Authors' contributions

MT carried out the molecular studies, drafted the manuscript, performed the both sequence and statistical analyses and participated in the design of the study. FP and AA participated in the design of the study and participated in the corrections. JD and MCR conceived of the study, participated in its design and in the corrections. All authors read and approved the final manuscript.

## Figures and Tables

**Table 1:** List of global analysis studies on the kidney

Study	Methods	Conditions	Species and tissue
		Injury or physiologic	
A. Global analysis studies in ARF			
Basile et al. (2005)	microarray	ischemia-reperfusion	Rat kidney
Kieran et al. (2003)	microarray	ischemia-reperfusion	Mouse kidney
Mishra et al (2003)	microarray	ischemia-reperfusion	Rat kidney
	microarray	cisplatin	Mouse kidney
Supavekin et al. (2003)	microarray	ischemia-reperfusion	Mouse kidney
Yoshida et al. (2002)	microarray	ischemia-reperfusion	Mouse kidney
Yoshida et al. (2002)	microarray	ischemia-reperfusion	Rat kidney
Huang et al. (2001)	microarray	cisplatin	Rat kidney
B. Global analysis providing control positive data to validate our SAGE libraries			
Chabardés-Garonne et al. (2003)	SAGE	normal	Human kidney
Cheval et al. (2002)	SADE*	normal	Mouse kidney
Elalouf et al (2002)	SADE*	normal	Mouse kidney
Schelling et al. (2002)	SAGE	normal	Mouse Kidney
Yano et al. (2002)	microarray	normal	Human kidney
El-Meanawy et al. (2000)	SAGE	normal/	Mouse kidney
		glomerulosclerosis-prone	
Virlon et al. (1999)	SADE*	normal	Mouse kidney

*SAGE Adaptation for Downsized Extracts

**Table 2:** SYBR Green (a) and Taq Man primers (b) sequences used for RT PCR reactions.

GeneSymbol	Accession No.	Primer 5' -> 3' sequence	Amplicon size (bp)
a. Primers using SYBR Green detection			
*Hprt*	NM_013556	Forward 5'-TTGCTGACCTGCTGGATTAC-3'	112
		Reverse 5'-CCCGTTGACTGATCATTACA-3'	
*Sod1*	XM_128337	Forward 5'-TGGTGGTCCATGAGAAACAA-3'	75
		Reverse 5'-TCCCAGCATTTCCAGTCTTT-3'	
*Ftl1*	NM_010240	Forward 5'-TGTGCAGAAGCCATCTCAAG-3'	99
		Reverse 5'-AGATCCAAGAGGGCCTGATT-3'	
*Opn*	NM_009263	Forward 5'-GACCATGAGATTGGCAGTGA-3'	110
		Reverse 5'-GGTGCAGGCTGTAAAGCTTC-3'	
*Odc*	NM_013614	Forward 5'-TTGCCACTGATGATTCCAAA-3'	129
		Reverse 5'-CATGGAAGCTCACACCAATG-3'	
*Calm2*	NM_007589	Forward 5'-CATGGCTGACCAACTGACTG-3'	103
		Reverse 5'-CCCCAGCTCCTTTGTTGTTA-3'	
*Igfbp7*	NM_008048	Forward 5'-GCATCCAGCCACCTTATGAT-3'	105
		Reverse 5'-ATCCGAAGAGGAGGAAGAGG-3'	
*Rps29*	NM_009093	Forward 5'-ACGGTCTGATCCGCAAATAC-3'	138
		Reverse 5'-CATGATCGGTTCCACTTGGT-3'	
*Tctp*	NM_009429	Forward 5'-CCGGGAGATCGCGGAC-3'	92
		Reverse 5'-TTCCACCGATGAGCGAGTC-3'	
b. Primers using Taq Man technology Assay ID (Assay On Demand, Applied Biosystems)			
*Hprt*	NM_013556	Mm00446968m1	
*Kap*	NM_010594	Mm00495104m1	
*Umod*	NM_009470	Mm00447649m1	
*NaPi-II*	NM_011392	Mm00441450m1	

**Table 3:** List of tags with significant variation in expression level induced by UN injection (p < 0.05). The table includes their frequency and their relevant accession number.

Tag sequence	Count		Gene name	Accession No.	Regulation (up: +, down: -)	Gene symbol
					
	UN-	UN+				
**Apoptosis**						
TGACACAGGA	4	0	cell death-inducing DNA fragmentation factor, alpha subunit-like effector B	NM_009894	-	*Cideb*
TAGGGCAATC	2	12	SMT3 (supressor of mif two, 3) homolog 2, pseudogene 4		+	*Smt3 h2-ps4*
**Amino acid metabolism**						
ATTAACTTGG	4	16	glutamate dehydrogenase	NM_008133	+	*Glud*
ATACTAACGT	53	14	ornithine decarboxylase, structural	NM_013614	-	*Odc*
**Carbohydrate metabolism**						
TGGATGCCTT	7	1	alcohol dehydrogenase 1, complex	NM_007409	-	*Adh1*
TGGATCTCTG	5	0	carbonic anhydrase 4	NM_007607	-	*Car4*
CTATAATGCC	25	8	fructose bisphosphatase 1	NM_019395	-	*Fbp1*
TTGTTCTGAC	0	6	glucose phosphate isomerase 1 complex	NM_008155	+	*Gpi*
CAGTTTGTAA	4	0	pyruvate dehydrogenase E1 alpha 1	NM_008810	-	*Pdaha*1
ATAAGGGATT	0	6	UDP-galactose translocator 2	NM_016752	+	*Ugalt2*
**Cell adhesion**						
GAAGAAAGGG	10	1	platelet/endothelial cell adhesion molecule	NM_008816	-	*Pecam*
**Cell cycle/mitosis**						
GCGAGGGAGC	4	0	dim1 (S. pombe)	NM_025299	-	Dim-pending
TGCTGCCATT	0	6	tubulin alpha 6	NM_009448	+	*Tuba6*
**Homeostasis**						
CCCTGGGTTC	32	62	ferritin light chain 1	NM_010240	+	*Ftl1*
**Immunity and defense**						
*Immunity*						
TTATGGAAT	0	7	CD24a antigen	NM_009846	+	*Cd24a*
GTTCAAGTGA	1	9	Ia-associated invariant chain Ag CD74 classe II	NM_010545	+	*Ii/CD74*
TAGGGAATGG	0	5	interferon-induced protein with tetratricopeptide repeats 3	NM_010501	+	*Ifit3*
CTGAGAGATA	1	13	lectin, galactose binding, soluble 3	NM_010705	+	*Lgals3/Gal-3*
ATTGGGGGAG	6	18	osteoclast inhibitory lectin	NM_053109	+	*ocil-pending*
CAAACACCGT	7	104	secreted phosphoprotein 1	NM_009263	+	*Spp1, Opn*
TGGGTTGTCT	149	263	translationally regulated transcript (21 kDa),	NM_009429	+	*Trt, Tpt1, Tctp*
TTTTATGTTT	12	21	tumor necrosis factor, alpha-induced protein 1 (endothelial)	NM_009395	+	*Tnfaip1*
TATACATCCA	23	69	uromodulin	NM_009470	+	*Umod*
*Antioxidant and free radical removal*						
CTATCCTCTC	419	537	glutathione peroxidase 3	NM_008161	+	*Gpx3*
ATGTGGTGTG	29	15	peroxiredoxin 1	NM_011034	-	*Prdx1*
GGTGAGCCTG	1	8	peroxiredoxin 2	NM_011563	+	*Prdx2*
TATCTGTGCA	28	16	selenoprotein P, plasma, 1	NM_009155	-	*Sepp*1
AGAAACAAGA	19	6	superoxide dismutase 1, soluble	XM_128337	-	*Sod1*
TTGCTTCTAT	12	0	thioether S-methyltransferase	NM_009349	-	*Temt*
CTACGTTCTC	2	9	thioredoxin-like 2	NM_023140	+	*Txnl2*
**Lipid fatty acid and steroid metabolism**						
AGCCCTCGGA	5	0	acetyl-Coenzyme A dehydrogenase, short chain	NM_007383	-	Acads
TTAAGACCTG	10	21	crystallin, zeta	NM_009968	+	*CryZ*
TAGAATAAAC	4	0	cytochrome P450, 2d9	NM_080006	-	*Cyp2d9*
TGTGTGGAAT	16	7	cytochrome P450, subfamily IV B, polypeptide 1	NM_007823	-	*Cyp4b1*
TGCCAACGCA	25	10	hydroxysteroid dehydrogenase-4	NM_008292	-	*Hsd17b4*
AATTTAATTA	5	0	phytanoyl-CoA hydroxylase	NM_010726	-	*Phyh*
**Muscle contraction**						
TGCAGTCATC	8	1	transgelin	NM_011526	-	*Tagln*
CTCTGGGGCT	0	6	transgelin 2	NM_178598	+	*Tagln2*
**Nucleoside, nucleotide and nucleic acid metabolism**						
CTAGATTGCG	4	0	ectonucleoside triphosphate diphosphohydrolase 5	NM_007647	-	*Entpd5*
AAGAAACCAG	33	16	expressed in non-metastatic cells 2, protein (NM23B) (nucleoside diphosphate kinase)	NM_008705	-	*Nme2*
GTGTAATAAG	2	8	heterogeneous nuclear ribonucleoprotein A2/B1	NM_016806	+	*Hnrpa2b1*
GGGAGCGAAA	16	8	inhibitor of DNA binding 2	NM_010496	-	*Idb2*
TACTGTCCTG	5	0	RNA polymerase 1–3 (16 kDa subunit)	NM_009087	-	*Rpo1–3*
**Oxydoreductase**						
AATGGCTAGC	13	5	cytochrome c, somatic	NM_025567	-	*Cycs*
**Protein metabolism and modification**						
*Amino acid activation*						
GCCAATAGTG	0	5	endothelial monocyte activating polypeptide 2	NM_007926	-	*Emap2/Scye1*
*Protein biosynthesis*						
TCACCCAATA	64	44	eukaryotic translation elongation factor 2	NM_007907	-	*Eef2*
CTAATAAAGC	34	68	Finkel-Biskis-Reilly murine sarcoma virus (FBR-MuSV) ubiquitously expressed (fox derived)	NM_007990	+	*Fau*
AGATCTATAC	13	4	ribosomal protein L7	NM_011291	-	*Rpl7*
AACAATTTG	21	52	ribosomal protein L9	NM_011292	+	*Rpl9*
GGCAAGCCCC	20	42	ribosomal protein L10A	NM_011287	+	*Rpl10a*
TGGATCAGTC	77	131	ribosomal protein L19	NM_009078	+	*Rpl19*
ATTCTCCAGT	13	36	ribosomal protein L23	NM_022891	+	*Rpl23*
CCCACAAGGT	16	6	ribosomal protein L27	NM_011289	-	*Rpl27*
GGCTTCGGTC	55	124	ribosomal protein, large, P1	NM_018853	+	*Rplp1*
TGTAGTGTAA	26	52	ribosomal protein S8	NM_009098	+	*Rps8*
TGGCCCAAA	2	12	ribosomal protein S16	NM_013647	+	*Rps16*
GCCTTTATGA	24	14	ribosomal protein S24	NM_011297	+	*Rps24*
TAAAGAGGCC	21	48	ribosomal protein S26	NM_013765	+	*Rps26*
CTAGTCTTTG	35	81	ribosomal protein S29	NM_009093	+	*Rps29*
*Protein folding*						
TAACAGTTGT	5	16	calnexin	NM_007597	+	*Canx*
GATTGTCAAA	1	11	FK506 binding protein 3 (25 kD)	NM_013902	+	*Fkbp3*
AGAGTTCAGA	13	4	heat shock protein, 60 kDa	NM_010477	-	*Hspd1/Hsp60*
GAATAATAAA	52	29	heat shock 70 kD protein 8	NM_031165	-	*Hspa8/Hsp73*
*Proteolysis*						
CCTTGCTCAA	11	27	cystatin C	NM_009976	+	*Cst3*
GAGAGTGTGA	13	5	kidney-derived aspartic protease-like protein	NM_008437	-	*Kdap*
ACCATTATAA	14	7	lysosomal membrane glycoprotein 2	NM_010685	-	*Lamp2*
TAGCTTCCTC	15	37	sequestosome 1	NM_011018	+	*Sqstm1/p62*
TGACCCCGGG	51	88	ubiquitin A-52 residue ribosomal protein fusion product 1	NM_019883	+	*Uba52*
CAGATCTTTG	6	19	ubiquitin C	NM_145578	+	*Ubc*
*Translation regulation*						
TGAAATACTT	31	15	heat-responsive protein 12	NM_008287	-	*Hrsp12*
**Signal transduction system, receptor**						
GCACAACTTG	3	15	calmodulin 2	NM_007589	+	*Calm2*
GGCTGTTGAA	0	10	cysteine rich protein	NM_007791	+	*csrp1*
CATACGCATA	15	3	growth hormone receptor	NM_010284	-	*Ghr*
GCTGCAGTTG	0	5	guanine nucleotide binding protein, beta 2, related sequence 1	NM_008143	+	*Gnb2-rs1*
AGAGACAAGG	23	4	N-myc downstream regulated 1	NM_006096	-	*Ndrg1*
ATCTCCTAGC	0	10	matrix gamma-carboxyglutamate (gla) protein	NM_008597	+	*Mglap*
AGCACTTTGC	1	10	Mus musculus, serine threonine kinase pim3, clone MGC:27707 IMAGE:4924687			+
GGCAGATGGA	17	8	parathyroid hormone receptor, PTH R	NM_011199	-	*Pthr*
TGGGGAAGAG	1	8	phosphatidylethanolamine binding protein	NM_018858	+	*Pbp*
TAACCAATCA	7	19	RAB5C	NM_004583	+	*Rab5c*
AAAATTGCTT	14	0	tyrosine 3-monooxygenase/tryptophan 5-monooxygenase activation protein, zeta polypeptide	NM_011740	-	*Ywhaz*
**Transport**						
*Electron transport*						
GCTTTGAATG	40	19	ATPase inhibitor	NM_007512	-	*Atpi*
GAAGGAAACC	4	0	ATP synthase, H+ transporting mitochondrial F1 complex, beta subunit	NM_016774	-	*Atp5b*
GCATACGGC	5	12	ATP synthase, H+ transporting, mitochondrial F1F0 complex, subunit e	NM_007507	+	*Atp5k*
TTATGAAATG	31	58	NADH dehydrogenase (ubiquinone) 1 alpha subcomplex, 1	NM_019443	+	*Ndufa1*
CTTGCAAGTG	40	26	NADH dehydrogenase (ubiquinone) 1 beta subcomplex, 9	NM_023172	-	*Ndufb9*
*Ion transport*						
ATGGGATTGA	0	21	glutamate receptor, ionotropic, kainate 1	NM_146072	+	*Grik1*
AGAGGCTACA	4	0	solute carrier family 21 (organic anion transporter)mb1	NM_013797	-	*Slc21a1*
GACACATACC	57	23	solute carrier family 22 (organic cation transporter)-like 2	NM_009203	-	*Slc22al2*
ACATTTCCAG	5	0	solute carrier family 27 (fatty acid transporter), mb 2	NM_011978	-	*Slc27a2*
TTGTGAGCCA	23	8	solute carrier family 34 (sodium phosphate), member 1	NM_011392	-	*Slc34a1*
*Lipid and fatty acid transport*						
GCTCTGATAC	5	0	sterol carrier protein 2, liver	NM_138508	-	*Scp2*
**Others**						
GAACCTCTCC	0	5	amyotrophic lateral sclerosis 2 (juvenile) homolog (human)	NM_028717	+	Als2
GAAGATCTAA	5	0	DNA segment, Chr 10, Johns Hopkins University 81 expressed	NM_138601	-	*D10jhu81e*
GTCGTGCCAT	59	12	DNA segment, Chr 7, Roswell Park 2 complex, expressed	NM_033080	-	*D7rp2e*
TCATTCTCCA	9	0	erythrocyte protein band 4.1-like 4a	NM_013512	-	*Epb4.1l4a*
CTCCTGCAGC	18	41	esterase 10	NM_016903	+	*Es10*
CTTCTTTCTG	0	7	growth arrest specific 6	NM_019521	+	*Gas6*
TAGCTTTAAA	45	85	insulin-like growth factor binding protein 7	NM_008048	+	*Igfbp7*
GACTTCACGC	283	91	kidney androgen regulated protein	NM_010594	-	*Kap*
GCATAGAAAT	22	8	kidney-specific membrane protein	NM_020626	-	*Nx17-pending*
TAACTGACAA	4	20	metallothionein 2	NM_008630	+	*Mt2*
TGACCACGGG	1	9	myeloid differentiation primary response gene 88	NM_010851	+	*Myd88*
GCCCCAATAA	20	12	phosphatidylcholine transfer protein-like	NM_019990	-	*Pctpl/Sdccagg28*
TAATACTCAA	73	49	upregulated during skeletal muscle growth 5	NM_023211	-	*Usmg5*
